# INNODIA Master Protocol for the evaluation of investigational medicinal products in children, adolescents and adults with newly diagnosed type 1 diabetes

**DOI:** 10.1186/s13063-022-06259-z

**Published:** 2022-05-18

**Authors:** David B. Dunger, Sylvaine F. A. Bruggraber, Adrian P. Mander, M. Loredana Marcovecchio, Timothy Tree, Piotr Jaroslaw Chmura, Mikael Knip, Anke M. Schulte, Chantal Mathieu, on behalf of the INNODIA consortium, C. Mathieu, P. Gillard, K. Casteels, L. Overbergh, D. Dunger, C. Wallace, M. Evans, A. Thankamony, E. Hendriks, S. Bruggraber, M. Peakman, T. Tree, N. Morgan, S. Richardson, J. Todd, L. Wicker, A. Mander, C. Dayan, M. Alhadj Ali, T. Pieber, D. Eizirik, M. Cnop, S. Brunak, F. Pociot, J. Johannesen, P. Rossing, C. Legido Quigley, R. Mallone, R. Scharfmann, C. Boitard, M. Knip, T. Otonkoski, R. Veijola, R. Lahesmaa, M. Oresic, J. Toppari, T. Danne, A. G. Ziegler, P. Achenbach, T. Rodriguez-Calvo, M. Solimena, E. Bonifacio, S. Speier, R. Holl, F. Dotta, F. Chiarelli, P. Marchetti, E. Bosi, S. Cianfarani, P. Ciampalini, C. de Beaufort, K. Dahl-Jørgensen, T. Skrivarhaug, G. Joner, L. Krogvold, P. Jarosz-Chobot, T. Battelino, B. Thorens, M. Gotthardt, B. Roep, T. Nikolic, A. Zaldumbide, A. Lernmark, M. Lundgren, G. Costecalde, T. Strube, A. Schulte, A. Nitsche, M. von Herrath, J. Wesley, A. Napolitano-Rosen, M. Thomas, N. Schloot, A. Goldfine, F. Waldron-Lynch, J. Kompa, A. Vedala, N. Hartmann, G. Nicolas, J. van Rampelbergh, N. Bovy, S. Dutta, J. Soderberg, S. Ahmed, F. Martin, G. Agiostratidou, A. Koralova, R. Willemsen, A. Smith, B. Anand, V. Puthi, S. Zac-Varghese, V. Datta, R. Dias, P. Sundaram, B. Vaidya, C. Patterson, K. Owen, B. Piel, S. Heller, T. Randell, T. Gazis, E. Bismuth Reismen, J-C Carel, J-P Riveline, J-F Gautier, F. Andreelli, F. Travert, E. Cosson, A. Penfornis, C. Petit, B. Feve, N. Lucidarme, E. Cosson, J-P Beressi, C. Ajzenman, A. Radu, S. Greteau-Hamoumou, C. Bibal, T. Meissner, B. Heidtmann, S. Toni, B. Rami-Merhar, B. Eeckhout, B. Peene, N. Vantongerloo, T. Maes, L. Gommers, M.L. Marcovecchio, J. Vela, E. Latres

**Affiliations:** 1 Department of Paediatrics, University of Cambridge, Cambridge, UK; 2 Wellcome Trust-MRC Institute of Metabolic Sciences, University of Cambridge, Cambridge, UK; 3 Centre for Trials Research, Cardiff University, Cardiff, UK; 4 NIHR Biomedical Research Centre, Guy’s and St Thomas’ NHS Foundation Trust and King’s College London, London, UK; 5 Novo Nordisk Foundation Center for Protein Research, Faculty of Health and Medical Sciences, University of Copenhagen, Copenhagen, Denmark; 6 Pediatric Research Centre, Children’s Hospital, University of Helsinki and Helsinki University Hospital, Helsinki, Finland; 7 Bayer AG, Berlin, Germany; 8 Department of Endocrinology, KU Leuven, Leuven, Belgium

**Keywords:** Beta-cell function, C-peptide, Master protocol, Phase 2, Prevention, Trials, Type 1 diabetes

## Abstract

**Background:**

The INNODIA consortium has established a pan-European infrastructure using validated centres to prospectively evaluate clinical data from individuals with newly diagnosed type 1 diabetes combined with centralised collection of clinical samples to determine rates of decline in beta-cell function and identify novel biomarkers, which could be used for future stratification of phase 2 clinical trials.

**Methods:**

In this context, we have developed a Master Protocol, based on the “backbone” of the INNODIA natural history study, which we believe could improve the delivery of phase 2 studies exploring the use of single or combinations of Investigational Medicinal Products (IMPs), designed to prevent or reverse declines in beta-cell function in individuals with newly diagnosed type 1 diabetes. Although many IMPs have demonstrated potential efficacy in phase 2 studies, few subsequent phase 3 studies have confirmed these benefits. Currently, phase 2 drug development for this indication is limited by poor evaluation of drug dosage and lack of mechanistic data to understand variable responses to the IMPs. Identification of biomarkers which might permit more robust stratification of participants at baseline has been slow.

**Discussion:**

The Master Protocol provides (1) standardised assessment of efficacy and safety, (2) comparable collection of mechanistic data, (3) the opportunity to include adaptive designs and the use of shared control groups in the evaluation of combination therapies, and (4) benefits of greater understanding of endpoint variation to ensure more robust sample size calculations and future baseline stratification using existing and novel biomarkers.

## Background

Type 1 diabetes is an autoimmune condition characterised by immune-mediated destruction of pancreatic beta-cells, leading to lifelong dependency on insulin therapy [1]. The pathogenic mechanisms leading to beta-cell loss may begin soon after birth, and by the time of type 1 diabetes diagnosis, residual beta-cell mass and function may be reduced by around 50% [2, 3].

Preservation of beta-cell function can improve glycaemic control, protect against hypoglycaemia and reduce the risk of long-term complications of type 1 diabetes [4, 5]. Several agents, either reflecting genetically validated pathways associated with the risk of type 1 diabetes [6–8] or those repositioned following documented efficacy in other autoimmune conditions [9], have been explored in an attempt to arrest the immune-mediated beta-cell destruction in type 1 diabetes. Alternative strategies have included the development of targeted immunomodulation such as oral insulin, DiaPep 277, GAD65, which have fewer side effects, but are probably more suitable for evaluation at an earlier stage of the life course of type 1 diabetes, to prevent progression from genetic susceptibility to the development of islet-related autoantibodies or from autoantibody positivity to the development of symptomatic type 1 diabetes [9, 10].

The results of the many immunotherapy studies undertaken in individuals newly diagnosed with type 1 diabetes have been recently reviewed [10]. Many of the agents which showed apparent efficacy in phase 2 failed to be confirmed in phase 3, perhaps emphasising the heterogeneity of response which could have been predicted by initial patient selection or more detailed analysis of predicted immunological responsiveness. Nevertheless, such studies confirm that these immunological interventions can, in some cases, provide clinically significant preservation of beta-cell function, but this is rarely sustained over time [10].

The design of these studies has been informed largely from data arising from TrialNet and other studies on longitudinal changes in C-peptide, as assessed by mixed meal tolerance tests (MMTT), over the first 2–3 years from diagnosis [11]. Following observed improvements in C-peptide during phase 2 studies, phase 3 studies, often involving over 300–500 subjects and taking more than 5 years before results are reported, have not yet led to drug approval for this indication. Furthermore, subsequent comparison of the results across studies can be difficult because of obvious variance in control groups, lack of comparable mechanistic evaluations and, in the case of commercial studies, lack of access to metadata and biological samples.

Thus, there is a lack of reference standards to assess comparability of these studies. Furthermore, a natural interpretation of the study results indicates that stratification of participants and standardisation of mechanistic efficacy and safety outcomes are essential. This will become even more critical with the suggestion that combination therapies, consisting of immunomodulation along with attempts to stimulate beta-cells, are evaluated [12]. The INNODIA Master Protocol was designed to address these issues by improving the speed and consistency of phase 2 studies for this indication.

## Methods/design

The INNODIA Master Protocol is built on the backbone of the ongoing INNODIA natural history study, which enables standardised recruitment, centralised data collection and assessment of changes in beta-cell function in relation to established and exploratory immune/metabolic biomarkers in newly diagnosed individuals with type 1 diabetes identified within 6 weeks from diagnosis across Europe.

### INNODIA natural history study

INNODIA is a global partnership between 31 academic institutions, six industrial partners, a small- and medium-sized enterprise and two leading funding organisations (Juvenile Diabetes Research Foundation and The Leona M and Harry B Helmsley Charitable Trust), bringing their knowledge and experience together with one common goal: “To fight Type 1 diabetes” (https://www.innodia.eu/). The overall aim of INNODIA is to advance in a decisive way, how to predict, stage, evaluate and prevent the onset and progression of type 1 diabetes. For this, INNODIA has established a comprehensive and interdisciplinary network of clinical and basic scientists, who are leading experts in the field of type 1 diabetes research in Europe, with complementary expertise from the areas of immunology, beta-cell biology, biomarker research and type 1 diabetes therapy. Joining forces in a coordinated fashion with industry partners and two charitable foundations, as well as with all major stakeholders, including regulatory bodies and patients with type 1 diabetes and their families. The project, approved in November 2015 and launched in January 2016, runs under the framework of the Innovative Medicines Initiative – Joint Undertaking [13] with a dedicated governance structure ensuring close interaction, communication and adherence to the objectives and deliverables of the consortium.

#### Development of centralised protocols and procedures

Extensive preliminary work involving partners from several academic institutions led to the development of an agreed protocol to enable studies of the relationship between changes in beta-cell function, immune profiles and genetic and environmental factors, in individuals with new-onset type 1 diabetes. The INNODIA natural history study protocol’s latest version is available on ClinicalTrials.gov (NCT03936634).

Protocol-specific standard operating procedures (SOPs) for sample collection and processing have been established. An electronic case report form (eCRF) documenting patient recruitment, evaluation, follow-up and sample management tracking and analysis has been developed and implemented throughout the INNODIA clinical network. The protocol and all the associated tools have been successfully used in the INNODIA natural history study, approved first in the UK in October 2016 and subsequently in 12 other countries in Europe by early 2018. To date, the network has consented more than 500 participants with new-onset type 1 diabetes to the natural history study (https://www.innodia.eu/).

#### Data handling, storage and data security

INNODIA has established its own data warehouse for the recording and safe keeping of all the data generated by the consortium, via the in-built online eCRF. A Data Management and Access Plan, detailing a clear set of policies for how these data and samples are collected and stored within INNODIA and how they are transferred between researchers, is in place.

All data, including backups, are encrypted and logically replicated for disaster recovery purposes. The infrastructure hosting the eCRF platform and the data warehouse for INNODIA is located in the Danish National Supercomputing Centre, which has been designed and built to store and process personal-sensitive data in accordance with the latest European regulations.

Clinical sites are required to comply with current Good Clinical Practice (GCP) and data protection policies. The principal investigators at each site take the responsibility to ensure the local teams follow the protocol and study manual. A delegation log and staff training log assure monitoring of clinical team compliance. In addition to the study protocol, reviewed and approved by ethics committees in each participating site/country, a study manual of operation and a series of SOPs for collection, storage and shipment of biosamples, such as whole blood, plasma, serum, urine and stool, have been developed. The manual re-states the rules for eligibility, recruitment and visit schedule, as per protocol, and also integrates specific rules for assessments, such as MMTT, type of material to be used and data to be collected.

INNODIA partners with extensive experience in the analysis of type 1 diabetes biomarkers for research projects at national or international levels form the INNODIA central laboratories where analysis of C-peptide and diabetes autoantibodies, genotyping and immune-phenotyping (immune monitoring hub) on samples collected across the clinical network are performed.

The immune monitoring hub, consisting of six research laboratories across the network, has developed a wheel and spoke model for assay development, analysis and validation.

INNODIA partners with expertise in clinical studies and/or trials form the INNODIA clinical network. Altogether, they are responsible for the care of more than 15 thousand children and adults with type 1 diabetes, making INNODIA the largest type 1 diabetes clinical network in Europe.

#### INNODIA governance structure

Due to the complexity of the INNODIA consortium and the number of partners involved, a governance structure has been designed which ensures a balanced representation of all participants involved from both the public consortium and industry partners. Efficient, flexible and professional day-to-day management of the project is achieved through the formation of a dedicated Coordination Team supported by a dedicated Project Management workpackage, ensuring the administrative and financial coordination of the project. This includes the instalment of all management bodies and the stipulation of the roles and responsibilities as well as the decision-making processes.

The INNODIA Managing Board is responsible for monitoring and evaluation of the overall scientific progress and timely achievement of deliverables and milestones. It ensures that all beneficiaries are regularly updated on the scientific progress and takes corrective action in order to ensure project progress, quality and adherence to ethical regulations.

The INNODIA General Assembly is made up of one representative of each INNODIA beneficiary and is responsible for the determination of policies and decision-making in relation to the overall strategy and progression of the consortium.

INNODIA also benefit from the support of a dedicated Patient Advisory Committee (PAC) to ensure that the views of patients and their families are incorporated within the study.

### Development of the INNODIA Master Protocol

**Fig. 1:**
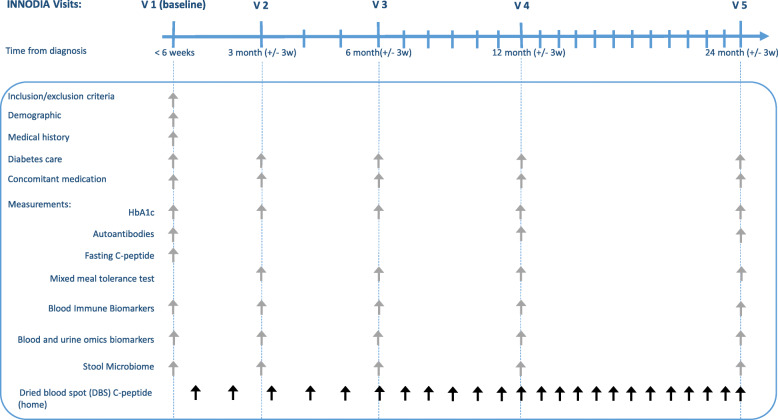
INNODIA Natural history study backbone

The INNODIA Master Protocol has been designed from the INNODIA natural history study using the same inclusion criteria (Table 1), visit schedule, and sample collection for standardised efficacy and mechanistic studies (Fig. 1). The INNODIA central laboratories, eCRF, central data warehouse and robust centralised governance structures described above, represent the “backbone” for the design of any future phase 2 studies described in the Master Protocol.

**Table 1:** Inclusion and exclusion criteria of the INNODIA natural history study

**Inclusion criteria**	To be included in the study, participants with newly diagnosed type 1 diabetes must: 1. Have given written informed consent to participate 2. Be aged between 1 and < 45 years 3. Be less than 6 weeks from the diagnosis of type 1 diabetes and requiring insulin treatment
**Exclusion criteria**	1. Non-type 1 diabetes (type 2 diabetes, monogenic diabetes and secondary diabetes) 2. Concurrent use of long-term immunosuppressive agents including oral steroids or medication likely to confound the interpretation of study results 3. Expected non-compliance with the protocol 4. Any medical history or clinically relevant abnormality that is deemed by the principal investigator and/or co-investigator to make the patient ineligible for inclusion because of problems in interpreting data or safety concerns 5. Participating in interventional or other drug research studies which could affect the primary objectives of the study

#### Adaptation for clinical trials

The existing INNODIA backbone and the centralised structures have been adapted in the development of the Master Protocol for use in phase 2 clinical trials across the INNODIA network using accredited clinical centres in Europe.

The essential inclusion and exclusion criteria are defined by the need to recruit individuals early following the diagnosis of type 1 diabetes and exclude those where the diagnosis may be in doubt (Table 1).

These criteria can be expanded where for example the efficacy of the Investigational Medicinal Product (IMP) could be dependent on a specific genotype (i.e. HLA DR3 or DR4) or other baseline characteristics (i.e. age group, BMI). The criteria can also be adapted for each sub-trial if based on the IMP, individuals with specific concomitant or previous medical conditions, or taking certain medications need to be excluded [14].

However, the clinical and mechanistic evaluations remain largely unchanged providing the primary outcomes and the potential to explore a more detailed mechanistic analysis of variability in response.

**Fig. 2:**
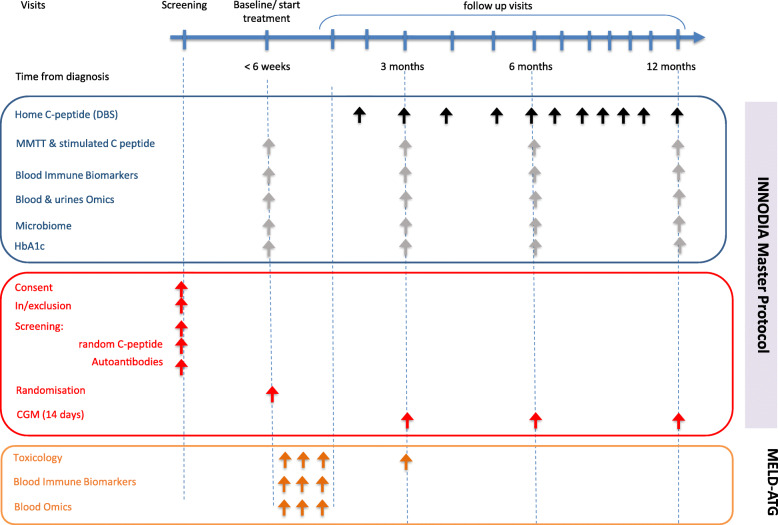
Adaption of the Master Protocol for a clinical trial (example MELD-ATG study)

The addition of study visits and sample collection points can permit essential evaluation of toxicology and pharmacokinetics/pharmacodynamics. An example of how the INNODIA backbone can be adapted for these additional studies is provided in Fig. 2, where the INNODIA baseline visit is combined with the trial screening visit, and early visits incorporate collection of essential safety and potential pharmacokinetics/pharmacodynamics data, as needed based on the specific IMP tested in each trial [14].

The INNODIA follow-up visits continue to provide standardised assessments and a backbone for answering state of the art primary and secondary objectives in trials in type 1 diabetes including monitoring of MMTTs and home-dried blood sample (DBS) collection to assess beta-cell function, changes in insulin dose, HbA1c and “time in range”, as assessed by continuous glucose monitoring (CGM). Extra visits related to drug safety and/or mechanistic time points can be added to the schedule whilst ensuring that standard mechanistic data are consistently collected.

Harmonised protocols and SOPs for sample collection, storage and analysis, established as part of the INNODIA natural history study, are used for all Trials following this Master protocol (Fig. 1). As shown in Figs. 1 and 2, samples will be collected for biochemical, immunological and omics studies and these include plasma, serum or whole blood specimens and urine and stool samples, which will be analysed either fresh or after storage, depending on the specific biomarkers.

Blood samples will be collected for the measurement of autoantibodies (glutamic acid decarboxylase antibodies-GADA, insulin autoantibodies-IAA, IA-2 antibodies-IA-2A or zinc transporter 8 antibody-ZnT8A), C-peptide and HbA1c. Additional blood samples will be collected for measuring immunological biomarkers. Blood, urine and stool samples will be also collected for analysis of omics biomarkers, which, depending on the specific trials, will include some or all of transcriptomics, miRNA, lipidomics, proteomics, metabolomics and microbiome.

#### Flexibility afforded by the Master Protocol

A recent review of master protocols [15] showed a surge in the number of platform trials, particularly within oncology. We introduce a new concept that is the INNODIA programme trial, which may consist of many sub-trials, the sub-trials can be platform trials, and each sub-trial may target different populations. Sub-trials can include different age ranges or be enriched using genetic markers and thus cannot be readily moulded into the perceived standard platform trial of a single trial population. Although the potential loss of statistical efficiency may be a consideration, there are still logistical efficiency gains and other statistical benefits of this INNODIA Master Protocol:

##### Sample size considerations

Often, trials are designed using estimates for the variance of the primary endpoint from an external study that sometimes has different inclusion and exclusion criteria to the one planned. One major benefit of having an observational cohort (INNODIA natural history study) with the same standardised measurements and SOPs as the planned trial is to therefore reliably use the observed data, from the INNODIA natural history study, to estimate the parameters needed for a sample size calculation such as the variance of the endpoint. Data from the INNODIA natural history study can also be used to estimate changes in the primary endpoint over the trial follow-up period to indicate whether planned treatment effects are reasonable within the specified target population and the likely changes on placebo. Additionally, the observational data could be used as historical controls and the design or analysis could use a Bayesian dynamic borrowing approach [16, 17] or one of several other approaches to borrowing information reviewed by Viele et al. [18].

##### Adaptive designs

The INNODIA programme trial has many outcomes that are repeated over the follow-up period, for example serial MMTTs (every 3–6 months) and DBS C-peptide (monthly) determinations. The repeated measures allow longitudinal adaptive designs, where interim analyses can use partial follow-up data to help make decisions. The observational cohort provides crucial estimates of the time courses and correlations between measurements that could remove the uncertainty when designing and calculating the operating characteristics of the planned adaptive designs. For example, a sub-trial may use a combination of the DBS C-peptide and early readouts from MMTTs to help make early decisions such as dropping treatment arms for futility, altering the allocation to more favourable treatments or altering the dose of an intervention. There is also a potential to discover biomarkers to use in future INNODIA trials that could lead to more sensitive outcomes or predictive strata.

##### Shared control groups

One strong potential benefit to both participants and trial designs is the use of shared controls. Multiple sub-trials, undertaken concurrently, have the benefit of sharing the same controls, if they are recruited when a trial is open. There is a direct saving because, if two trials use the same control group, they halve the sample size of the control arm for the single trial. In addition, participants are more likely to receive an active treatment rather than placebo, and hence, this should increase participants’ benefit (and arguably making a more ethical trial).

Finally, there is currently no consensus on the use of historical data even if they are part of the same platform as there may be population drift. The use of historical control data should pass the stringent conditions laid out by Pocock [19] and the Master Protocol ensures this within INNODIA.

For the INNODIA trials, control data will be primarily those obtained from placebo groups enrolled in other trials conducted within the INNODIA consortium. Potential historical controls are those recruited in the INNODIA natural history study. This implies that controls are recruited within the INNODIA network, in the same clinical/research centres than trial participants, and therefore from a similar background population and, by the same principal investigators and study teams, all trained and following INNODIA SOP. In addition, the same eligibility criteria and treatment evaluation will apply to controls and treatment groups.

##### Statistical analysis plans

The Master Protocol allows further standardisation of the statistical analyses across sub-trials. Although trial designs may differ by the number of intervention arms and may have different populations, often endpoints, such as C-peptide, can be analysed using the same mixed effects model with the same list of potential confounders. The relevant treatment effects can be extracted from the models. Given that many of the measurements are standardised, often summary tables, secondary analyses and exploratory analyses can be specified within what we have labelled the master Statistical Analysis Plan (SAP). A master SAP is a comprehensive document containing all the required outputs for the sub-trials and allows operational efficiencies and informative reports for all future sub-trials.

#### Regulatory, ethics and dissemination plans

Successful linkage of the INNODIA natural history eCRF to an interventional clinical trial has been recently achieved in the DiViDint trial (EudraCT Number: 2015-003350-41; Sponsor: Oslo University Hospital – The Diabetes Virus Detection and intervention trial) (Table 2).

**Table 2:** Current trials based on the INNODIA Master Protocol

**Trial reference**	**MELD-ATG** ClinicalTrials.gov Identifier: NCT04509791 EudraCT 2019-0013265-17 Sponsor: Universitair Ziekenhuis Leuven	**VER-A-T1D** ClinicalTrials.gov Identifier: NCT04545151 EudraCT 2010-000435-45 Sponsor: Medical University of Graz	I**MPACT** ClinicalTrials.gov Identifier: NCT04524949 EudraCT 2020-001317-20 Sponsor: Imcyse SA	**CFZ33 ISCALIMAB** ClinicalTrials.gov Identifier: NCT04129528 EudraCT 2018-004553-25 Sponsor: Novartis Pharma AG	**DiViDint** ClinicalTrials.gov Identifier: NCT04838145 EudraCT 2015-003350-41 Sponsor: Division of Paediatric and Adolescent Medicine, Oslo University Hospital
**Full title**	MELD-ATG: Phase II, Dose Ranging, Efficacy Study of Anti-thymocyte Globulin (ATG) Within 6 Weeks of Diagnosis of Type 1 Diabetes (T1D)	A Randomised, Double-blind, Placebo Controlled, Parallel Group, Multi-centre Trial in Adult Subjects With Newly Diagnosed Type 1 Diabetes Mellitus Investigating the Effect of Verapamil SR on Preservation of Beta-cell Function (Ver-A-T1D)	A Phase Ib/IIa, Randomized, Double-blind Placebo-controlled, Multicenter Adaptive Design Clinical Trial to Evaluate the Immune Signature of the Treatment With the Imotope IMCY-0098 and Its Effect on the Preservation of Beta-cell Function in Young Adult and Adolescent Patients With a Recent Onset Type 1 Diabetes	Investigator- and subject-blinded, randomized, placebo-controlled study to evaluate safety, tolerability, pharmacokinetics and efficacy of CFZ533 in pediatric and young adults with new-onset type 1 diabetes	The Diabetes Virus Detection and Intervention Trial
**Treatment arms**	7 cohorts, each recruited sequentially, with between 3 and 5 treatment arms	2 arms Active drug vs placebo	3 arms: IMCY-0098, low dose IMCY-0098, high dose Placebo	2 arms Active drug vs placebo	2 arms Active drug vs placebo
**Age group**	5–25 years	18–45 years	18–45 years	6–21 years	6–15 years
**Treatment modality**	Intravenous infusion for 2 consecutive days	Tablets: once daily (titrated 120 to 360 mg) for 1 year	Subcutaneous injection: 6 times fortnightly; booster dose at 24 weeks	Intravenous for first dose infusion, then home subcutaneous injections for 1 year	Oral solution: once daily for 26 weeks
**Total duration**	13 months	13 months	13 months	16–36 months	36 months

The INNODIA Clinical Trial Master Protocol was submitted to the European Medicines Agency Scientific Advice Working Party (SAWP) of the Committee for Medicinal Products for Human Use (CHMP) for Qualifications advice for novel methodologies in clinical drug development. A dossier was submitted and following written and face-to-face discussions with representatives from the SAWP, the final advice was received in February 2020 [EMA/CHMP/SAWP/13547/2020]. The advice received was supportive of the context of the use of the Master Protocol and the concept of standardisation of the investigative approach was endorsed.

**Fig. 3:**
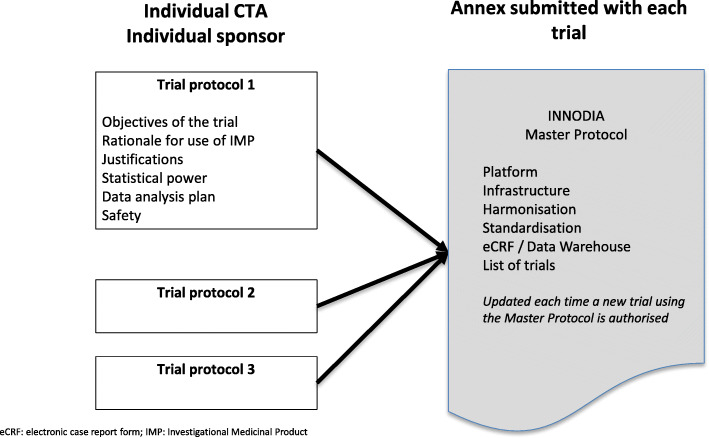
The regulatory framework for the INNODIA Master Protocol

Currently, several phase 2 clinical trials aligned to the Master Protocol have been initiated (Table 2). These studies will be linked to the INNODIA Master Protocol through submission of an annex to the approved Clinical Trials Authorisation. For each independent trial protocol, the trial sponsor will remain responsible for defining the objectives, the rationale and justification for the use of the IMP, statistical power calculations and data analysis plans and safety assessments. The Master Protocol annex will describe the underlying schedule of “backbone”, infrastructure, harmonisation, standardisation and, where applicable, the INNODIA eCRF and central data warehouse. The annex will be maintained using a SOP by the Clinical Trials Co-ordination team of INNODIA. They will be responsible for version control and updating details of all trials aligned to the Master Protocol and listing individual sites within each trial Protocol (Fig. 3).

The plan for the Master Protocol has been widely disseminated through the consortium annual plenums and is documented on INNODIA website (https://www.innodia.eu/). The development of the Master Protocol has been reported at oral sessions at the annual meetings of the International Society for Pediatric and Adolescent Diabetes and the European Association for the Study of Diabetes.

#### Patient and public involvement

The wider acceptability of our approach in people with type 1 diabetes has been evaluated by the INNODIA PAC. The INNODIA PAC has been directly involved in open discussions with the INNODIA clinical researchers and drugs companies, which are going to use the Master Protocol design, and their views and suggestions were implemented into the Master Protocol.

## Discussion

The Master Protocol is based on the analytic backbone that has been established in the INNODIA natural history study, with systematic assessment of beta-cell function, insulin dose, HbA1c, immunological studies and broad biomarker discovery work over the first 2 years from the diagnosis of type 1 diabetes. The Master Protocol will utilise the extensive established infrastructure within INNODIA which has already been successfully used to longitudinally study more than 500 participants with newly diagnosed type 1 diabetes, providing standardised assessment of efficacy and safety and comparable collection of mechanistic data. The Master Protocol based on this established backbone of patient selection and standardised assessments provides extensive flexibility to explore, at an early stage of drug development, the mechanism, safety, efficacy, dosing, of promising new medications proposed for delaying the progression of newly diagnosed type 1 diabetes.

The Master Protocol also offers the opportunity to include adaptive designs and the use of shared control groups in the evaluation of therapies and combinations of therapies, along with the benefits of greater understanding of variable endpoints to ensure robust sample size calculations and in the future, baseline stratification or enrichment by existing and novel biomarkers.

## Trial status

Currently, several phase 2 clinical trials aligned to the Master Protocol have been initiated (Table 2).

## Abbreviations

CGM: Continuous glucose monitoring
DBS: Dried blood spot
eCRF: Electronic case report form
HbA1c: Glycated haemoglobin
IMP: Investigational medicinal product
INNODIA: An innovative approach towards understanding and arresting type 1 diabetes
MMTT: Mixed meal tolerance test
SAP: Statistical analysis plan
SOP: Standard operating procedure
